# Noninvasive dynamic vascular imaging: arterial spin labeling-based noncontrast magnetic resonance digital subtraction angiography for cerebral disease diagnoses

**DOI:** 10.1007/s11604-025-01758-w

**Published:** 2025-03-12

**Authors:** Miho Gomyo, Kazuhiro Tsuchiya, Kenichi Yokoyama

**Affiliations:** Department of Radiology, Kyorin University Faculty of Medicine, 6-20-2 Shinkawa, Mitaka, Tokyo 181-8611 Japan

**Keywords:** Arterial spin labeling, Magnetic resonance digital subtraction angiography, Pseudocontinuous ASL, Superselective labeling pulse, Ultrashort echo, High-temporal resolution

## Abstract

Digital subtraction angiography (DSA) is the gold standard for assessing cerebrovascular hemodynamics. DSA is predominantly utilized to evaluate the hemodynamic information of various cerebral diseases. However, DSA is relatively invasive and involves radiation exposure and risks of allergic reactions or renal dysfunction related to iodine-based contrast agents. Magnetic resonance DSA (MRDSA) with a gadolinium-based contrast agent is a popular method for assessing hemodynamics without radiation exposure. However, it has the disadvantage of the risk of allergy due to the contrast agent and lower temporal and spatial resolution than DSA. In recent years, arterial spin labeling-based noncontrast MRDSA (ASL-MRDSA), which utilizes blood labeled with radiofrequency pulses as an endogenous tracer, has been developed. ASL-MRDSA exhibits the same temporal resolution as DSA; therefore, it can yield dynamic information about various cerebral diseases. Pulsed ASL has been used as the major labeling method. However, the recent development of the pseudocontinuous ASL method has enabled high ASL signal maintenance even at delayed phases. This advancement allows for a more detailed assessment of hemodynamic information about vascular diseases, including major artery severe stenosis, occlusion, and moyamoya disease. Furthermore, combining it with superselective labeling pulses also allows for a more detailed hemodynamic assessment of complex vascular malformations such as DSA. In addition, ASL-MRDSA using ultrashort-echo time can acquire signals before phase dispersion occurs, which reduces artifacts induced by metallic materials and blood flow. This technique is useful for assessing post-clipping, post-coiling, and pseudostenosis. Furthermore, ASL-MRDSA using a wheel scan mode without full sampling achieves higher temporal resolution than DSA, which enables blood flow evaluation within giant cerebral aneurysms. Herein, we introduce the basic principles and various novel techniques of ASL-MRDSA and demonstrate its clinical applications, pitfalls, and limitations in cerebral disease diagnoses.

**Supplementary Information:**

The online version contains supplementary material available at 10.1007/s11604-025-01758-w.

## Introduction

Digital subtraction angiography (DSA) is the gold standard for detailed cerebrovascular hemodynamics assessment. It has been predominantly used to investigate the hemodynamic information of various cerebral diseases, such as the assessment of hemodynamics and collateral circulation in cerebral arterial stenosis or occlusion, the pre- and post-treatment evaluation of various vascular lesions, including cerebral aneurysm, arteriovenous malformation (AVM), and arteriovenous fistula (AVF), and the preoperative examination of brain tumors. However, DSA is a relatively invasive procedure with related risks, including arterial dissection, ischemic stroke, and iodine-based contrast agent-induced allergic reactions or renal dysfunction [1, 2]. Further, concerns exist regarding increased radiation exposure during prolonged assessments. On the other hand, contrast-enhanced magnetic resonance DSA (MRDSA), which uses high-speed scanning techniques to conduct sequential data acquisition during rapid intravenous injection of gadolinium-based contrast agents, has been widely known as a method for assessing hemodynamics of cerebral diseases using MR imaging (MRI). Contrast-enhanced MRDSA involves no radiation exposure, but it has the disadvantage of the risk of toxicity and adverse effects related to the gadolinium-based contrast agents and lower temporal and spatial resolution than DSA [3–5]. Conversely, noncontrast-enhanced MRDSA based on arterial spin labeling (ASL-MRDSA) has been developed in recent years. ASL-MRDSA exhibits comparable temporal resolution as DSA and yields dynamic information on various cerebral diseases without a gadolinium-based contrast agent [6]. Moreover, the technology of ASL-MRDSA has been evolving in recent years. This review presents the principles and various scanning methods of ASL-MRDSA from conventional to novel techniques and demonstrates its clinical applications, pitfalls, and limitations in cerebral disease diagnoses.

## Basic principles of the ASL-MRDSA

The magnetically labeled spins of water molecules in the arterial blood are used as an endogenous tracer in ASL. Initially, selective radio frequency (RF) pulses are applied to the neck area to invert the longitudinal magnetization of the spins of water molecules in the arterial blood, thereby magnetically labeling the blood in the cervical vessels. Subsequently, signals are acquired when the labeled blood reaches the head area via blood flow. Then, ASL-MRA is generated by subtracting the unlabeled control images from the labeled images. ASL-MRDSA is obtained by repeating the data acquisition at different time points following the labeling. The labeling and signal acquisition methods of ASL-MRDSA and their characteristics are further described below.

## Readout sequences

The main readout sequence of ASL-MRDSA is based on the gradient-echo sequences, such as the turbo field-echo and spoiled gradient-echo. Signals in ASL-MRDSA are acquired, while the labeled spins fill the blood vessels, resulting in a high signal-to-noise ratio (SNR) within the blood vessels. Thus, a three-dimensional (3D) readout is now more predominantly used than a two-dimensional readout. All the ASL-MRDSA sequences introduced in this article utilize 3D acquisition. The 3D readout allows whole-brain scanning with small voxel sizes of approximately 1.0-mm isotropic voxels, causing high spatial resolution and enabling multidirectional observation.

## Readout acquisitions

ASL-MRDSA involves two main types of readout: single time point acquisition and multiphase acquisition. In the single time point acquisition, only one phase is acquired after the labeling pulse. Therefore, the labeling pulse and readout need to be repeated at any different post-labeling delay time to obtain multiple phases. Hence, the total scan time required to obtain multiple phases is significantly extended. On the other hand, multiphase acquisition is a method that uses a technique such as a look-locker readout to collect multiple phases after one labeling pulse and is currently used predominantly [7, 8]. However, the additional excitation RF pulse during readout in multiphase acquisition saturates the arterial signal within the imaging range and accelerates the T1 relaxation of the labeled blood. Thus, the ASL signal within the blood vessels decreases, which becomes more pronounced in the late phases.

## Labeling methods and characteristics

Three ASL labeling methods include pulsed ASL (PASL), continuous ASL (CASL), and pseudocontinuous ASL (PCASL). Labeling methods greatly influenced ASL signals. CASL is a method that utilizes continuous RF pulses and achieves a high SNR, but it has the disadvantage of significantly increasing the specific absorption rate, making its clinical application difficulty. Currently, the labeling methods utilized in ASL-MRDSA are PASL and PCASL. The following section describes these labeling methods and their characteristics in detail.

### (1) Pulsed ASL (PASL)

A single-short RF pulse is used in PASL to label a wide area of the cervical blood vessels with a typically 10–20 cm thick slab. The labeling pulse is less affected by vascular conditions, such as tortuosity, because of the thick slab. The typical duration of the labeling RF pulse is 5–20 ms. Therefore, the inflow of the labeled arterial blood into the intracranial vessels can be depicted by starting the readout shortly after the labeling pulse. Further, PASL uses a single-short RF pulse, causing shorter scan times and lower magnetization transfer effects than PCASL. Conversely, the labeling time is short, and the spins are inverted in the distal region of the labeling slab. Therefore, the spins undergo T1 decay while passing through the labeling slab, causing a low SNR and a short T1 relaxation time. The time from labeling to signal acquisition in PASL is referred to as the inversion time (TI). However, the T1 relaxation of the labeled blood accelerates as the TI extends. Moreover, the additional excitation RF pulses in multiphase acquisition during readout saturate the arterial signal and further accelerate the T1 relaxation of the labeled blood. Accordingly, ASL signals in the blood vessels markedly decrease in the late phases, and the signal decrease becomes more pronounced in slow blood flow areas. Each vendor provides ASL-MRDSA using PASL: 4D time-resolved angiography non-contrast-enhanced (4D-TRANCE; Philips Healthcare, Best, the Netherlands) and multi-TI MRA with ASTAR ASL technique (mASTAR; Canon Medical Systems, Tochigi, Japan). These temporal resolutions are approximately 250 ms. Acquisition frame rates in DSA are typically limited to reduce radiation exposure, and the current frame rate is approximately 3 or 4 frames per second [9], which corresponds to a temporal resolution of 250 or approximately 300 ms. Therefore, the temporal resolution of ASL-MRDSA is almost equivalent to that of DSA. In addition, the PASL method does not require any special hardware and is currently a popular ASL-MRDSA method.

### (2) Pseudocontinuous ASL (PCASL)

Multiple intermittent RF pulses are used in PCASL to label the cervical blood vessels. The slab thickness of the PCASL is thinner than that of the PASL, and the labeling slab needs to be set perpendicular to the blood vessel to achieve effective labeling. Therefore, it is more susceptible to vascular conditions such as tortuosity. Multiple intermittent RF pulses are utilized in PCASL; thus, the scan time is longer than that of PASL. The labeling bolus in PASL is determined by the thickness of the labeling pulse, whereas more labeled arterial blood can be generated in PCASL by applying labeling for a long duration, which allows the intravascular SNR to be kept higher than that in PASL. The time from labeling to signal acquisition in PCASL is referred to as the post-labeling delay (PLD). In recent years, 4D-MR angiography based on pseudo-continuous arterial spin labeling combined with CENTRA-keyhole and view-sharing (4D-PACK; Philips Healthcare) has been developed as ASL-MRDSA using PCASL [10]. The data acquisition of 4D-PACK is a single time point acquisition method that repeatedly performs labeling and data acquisition at different PLDs. Previously, PCASL with a single time point acquisition method required a long scan time, making its clinical practice application difficult. However, 4D-PACK has significantly reduced the scan time using CENTRA-keyhole [11] and view-sharing [12] technology. Obara et al. described the 4D-PACK scheme in detail [10]. The hemodynamic assessment of brain lesions has improved dramatically in recent years as PCASL with a single-time acquisition method has become clinically applicable.

## Selective labeling pulse method

The labeling slab can be set selectively to a target vessel. Generally, a thin labeling slab is utilized as the selective labeling pulse. However, a risk of reduced labeling efficiency occurs due to the thin labeling slab and the inclusion of other blood vessels in the labeling slab in the *z*-axis [13]. In recent years, the superselective labeling pulse has been enabled to be set only to the target vessel, allowing the assessment of the hemodynamics of each target vessel such as DSA. The novel technique that combines superselective labeling pulse with 4D-PACK is referred to as 4D-MR angiography based on super-selective pseudo-continuous arterial spin labeling combined with CENTRA-keyhole and view-sharing (4D-S-PACK; Philips Healthcar). 4D-S-PACK can assess the hemodynamics of only the target blood vessel and obtain good visualization of the peripheries even in the late phases [14].

### **Ultrashort-echo time sequence**

The ultrashort-echo time (UTE) sequence acquires data radially from the center of the k-space immediately after the RF excitation pulse and can perform a scan with an extremely short TE of almost zero. Therefore, the UTE sequence can acquire signals before phase dispersion, which significantly reduces the effect of phase dispersion. The UTE method is useful for cases susceptible to phase dispersion, including cerebral aneurysms after clipping, coil embolization with or without stent assist, and pseudoarterial stenosis related to tortuosity [15–21]. UTE-mASTAR and four-dimensional MRA with minimized acoustic noise utilizing ultrashort-echo time (mUTE-4D-MRA) (Canon Medical Systems) are provided as ASL-MRDSA using the UTE method.

## High-temporal resolution sequence

The use of a fast 3D mode [22], which is a wheel-type radial scan to mainly collect signals in the center of the k-space, may significantly reduce the scan time and phase interval time of ASL-MRDSA. ASL-MRDSA using this scan mode, high-temporal resolution mASTAR (Canon Medical Systems), enables scanning more phase numbers with higher temporal resolution. High-temporal resolution mASTAR achieves a much better temporal resolution of 87 ms and many phases up to 15. High-temporal resolution mASTAR exhibits a much higher temporal resolution than DSA. High-temporal resolution mASTAR shortens the signal acquisition time, compared to conventional ASL-MRDSA, enabling the acquisition of more phases in the same scan time and shortening the scan time with the same number of phases. However, the high-temporal resolution mASTAR uses a multiphase acquisition; thus, the arterial signals decrease in the late phase due to the RF saturation effect.

## Clinical applications

The clinical applications of ASL-MRDSA are presented below according to the clinical experience at our institution. All cases presented in this review article were investigated with the following 3T MRI scanners: Vantage Titan 3T, Vantage Galan 3T (Canon Medical Systems), and Ingenia Elition 3.0T X (Philips Healthcare). Tables 1 and 2 list the scan parameters of the ASL-MRDSA used at our institution. Table 1 shows the scan parameters of the conventional ASL-MRDSA, whereas Table 2 presents those of the novel technology. The TI, PLD, and phase numbers are set arbitrarily, and the scan time changes accordingly.

**Table 1:** The scan parameters of conventional ASL-MRDSA

Sequential name	4D-TRANCE	mASTAR	UTE-mASTAR
Vendor	Philips	Canon	Canon
Scanner	Ingenia Elition 3.0T X	Vantage Centurian	Vantage Titan
Field strength	3T	3T	3T
Readout sequence	3D-FFE	3D-FFE	3D-FFE
Readout acquisition	Multi-phase	Multi-phase	Multi-phase
Labeling method	PASL	PASL	PASL
Repetition time/echo time	9.4/4.9 ms	4.8/1.5 ms	3.7/0.096 ms
Slice thickness	1.5 mm	2.4 mm	1 mm
Number of slices	120	41	200
Phase interval (minimum settable value)	235 ms (160 ms)	235 ms (231 ms)	293 ms (293 ms)
Number of phases	8	6	4
First TI/last TI	435/2080 ms	435/1610 ms	300/1179 ms
Scan time	4 min 50 s	4 min 50 s	4 min 50 s

*ASL-MRDSA* arterial spin labeling-based noncontrast magnetic resonance digital subtraction angiography, *4D-TRANCE* 4D time-resolved angiography non-contrast-enhanced, *mASTAR* multi-TI MRA with ASTAR ASL technique, *UTE-mASTAR* ultrashort-echo time-multi-TI MRA with ASTAR ASL technique, *3D-FFE* 3D-first field echo, *PASL* pulsed arterial spin labeling, *TI* inversion time

**Table 2:** The scan parameters of novel technology

Sequential name	4D-PACK	4D-S-PACK	HTR-mASTAR
Vendor	Philips		Canon
Scanner	Ingenia Elition 3.0T X		Vantage Centurian
Field strength	3T		3T
Readout sequence	3D-FFE		3D-FFE
Acquisition technique	CENTRA-keyhole and view-sharing		Fast 3D mode
Labeling method	PCASL		PASL
Repetition time/echo time	7.3/3.2 ms	5.7/2.6 ms	4.8/1.5 ms
Slice thickness	1.5 mm	1.6 mm	2.4 mm
Number of slices	160	150	41
Post labeling time (PLD or TI)	200, 500, 900, 1400, 2000, 2600 ms	800, 1200, 1600, 2000, 2400, 2800 ms	200–1418 ms
Phase interval	300–600 ms	400 ms	87 ms
Number of phases	6	6	15
Scan time	8 min 19 s	7 min 15 s	5 min 43 s

4D-*PACK* 4D-MR angiography based on pseudo-continuous arterial spin labeling combined with CENTRA-keyhole and view-sharing, *4D-S-PACK* 4D-MR angiography based on super-selective pseudo-continuous arterial spin labeling combined with CENTRA-keyhole and view-sharing, *HTR-mASTAR* high-temporal resolution multi-TI MRA with ASTAR ASL technique, *3D-FFE* 3D-first field echo, *PLD* post-labeling delay, *TI* inversion time

## Cerebral arterial stenosis, obstruction, moyamoya disease, and post-bypass surgery

Collateral circulation supplements the blood flow in the hypo-perfused regions in progressive stenosis or occlusion of the major cerebral arteries such as atherosclerosis and moyamoya disease. When cerebral infarction occurs due to major artery occlusion, the stroke progression may be slow if the collateral circulation around the involved artery is well developed. In particular, mechanical thrombectomy may be indicated if the ischemia progression is slow. Leptomeningeal anastomosis (LMA) collateral impairment is a strong predictor of clinical outcomes in stroke patients with large-artery occlusion and endovascular treatment [23, 24]. Assessing the development of LMA collaterals will be useful in considering therapeutic interventions and predicting patient outcomes. In addition to the previous reasons, collateral circulation needs to be assessed because the degree of collateral circulation development varies among individuals. Three-dimensional time-of-flight MRA (TOF-MRA) is a useful noninvasive method that visualizes the morphology of cerebral arteries. However, arteries that are distal to severe stenosis and collateral circulation may be insufficiently visualized due to the decreased inflow caused by the RF saturation effect due to slow flow. This issue is particularly pronounced in vessels that run parallel to the imaging slab. Meanwhile, ASL-MRDSA is less susceptible to signal loss due to blood vessel directions, and background signals other than those of the labeled blood are suppressed by subtraction, resulting in improved peripheral artery visualization compared with TOF-MRA [25].

**Fig. 1:**
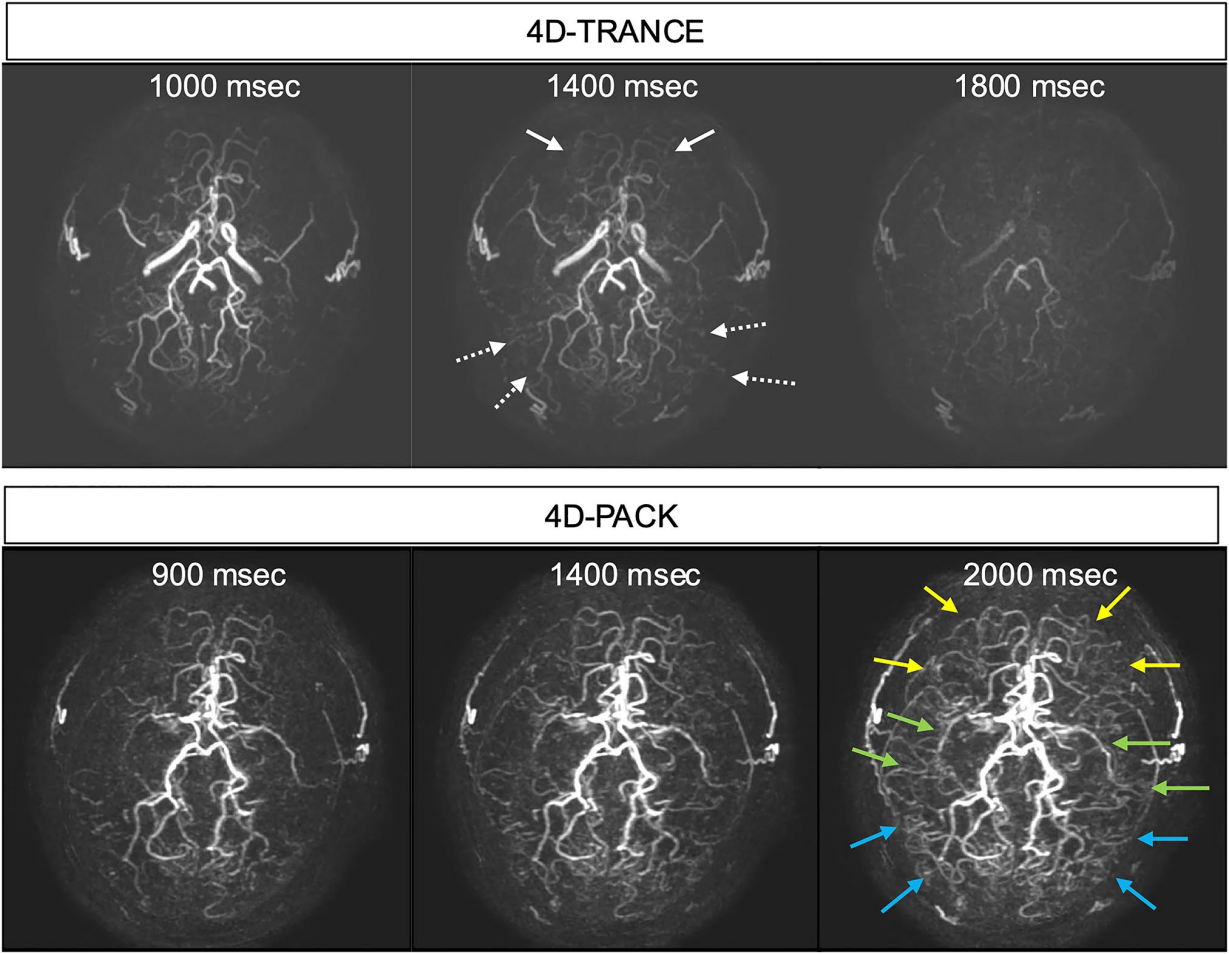
Moyamoya disease with severe stenosis of bilateral M1 origin. The inversion and the post-labeling times for PASL and PCASL are described above each figure. One of ASL-MRDSA with PASL, 4D-TRANCE, visualizes the collateral circulation from the anterior cerebral artery (arrows) and the posterior cerebral artery bilaterally (dotted arrows). Moreover, the novel ASL-MRDSA technique with PCASL, 4D-PACK, visualizes not only the collateral circulation from the bilateral anterior and posterior cerebral arteries (yellow and blue arrows) but also the peripheral branches distal to the severe stenosis of M1 (green arrows), which could not be depicted with PASL because of the radiofrequency saturation effects. 4D-TRANCE: 4D time-resolved angiography non-contrast-enhanced, 4D-PACK: 4D-MR angiography based on pseudo-continuous arterial spin labeling combined with CENTRA-keyhole and view-sharing, ASL-MRDSA: arterial spin labeling-based noncontrast magnetic resonance digital subtraction angiography, PASL: pulsed arterial spin labeling, PCASL: pseudocontinuous arterial spin labeling

**Fig. 2:**
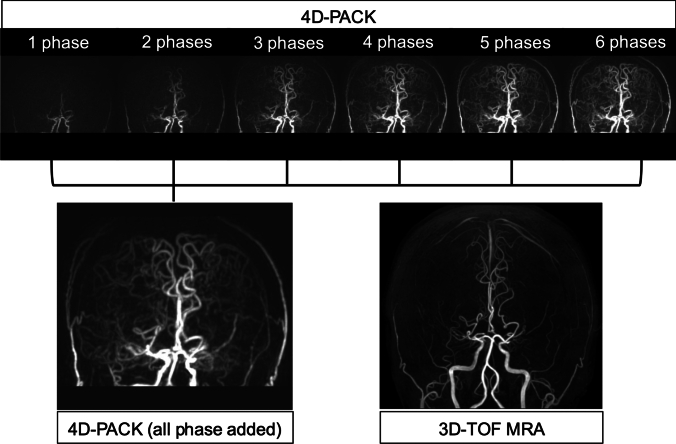
MIP image created by adding all ASL-MRDSA phases. This is the same case as Fig. 1. ASL-MRDSA enables the creation of MIP images that include all phases by adding all phase data. MIP images of 4D-PACK depict vessels, including peripheral branches from the bilateral M1 severe stenoses and the collateral circulations much better than 3D-TOF-MRA. 3D-TOF-MRA: 3-dimensional time-of-flight magnetic resonance angiography, 4D-PACK: 4D-MR angiography based on pseudo-continuous arterial spin labeling combined with CENTRA-keyhole and view-sharing, ASL-MRDSA: arterial spin labeling-based noncontrast magnetic resonance digital subtraction angiography, MIP: maximum intensity projection

**Fig. 3:**
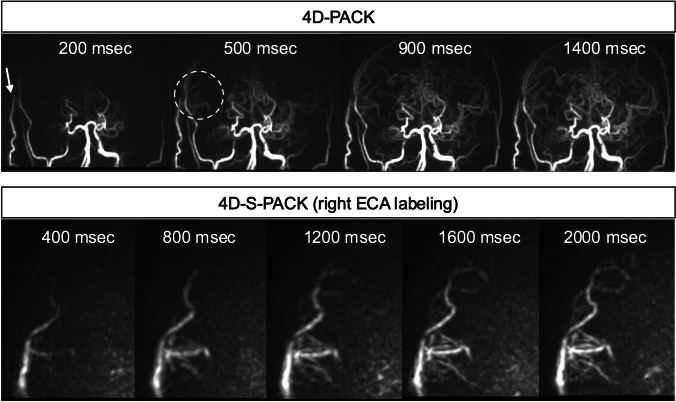
Post-right STA-MCA bypass. The upper column demonstrates the coronal views of 4D-PACK, and the lower column presents the coronal views of 4D-S-PACK with the right ECA labeling. 4D-PACK well visualizes bypass (arrow) and peripheral recipient arteries (dotted circle). Furthermore, using a superselective labeling pulse that is applied exclusively to the right STA reveals that most of the peripheral depictions of the recipient arteries are visualized via the bypass. 4D-PACK: 4D-MR angiography based on pseudo-continuous arterial spin labeling combined with CENTRA-keyhole and view-sharing, 4D-S-PACK: 4D-MR angiography based on super-selective pseudo-continuous arterial spin labeling combined with CENTRA-keyhole and view-sharing, ECA: external carotid artery, STA-MCA: superficial temporal artery to middle cerebral artery

In addition, ASL-MRDSA obtains time-axis information with the same temporal resolution as DSA, making the hemodynamic assessments of major cerebral arterial stenosis or occlusion possible. ASL-MRDSA is useful for evaluating hemodynamics in cases of major cerebral arterial stenosis or occlusion and those of moyamoya disease. ASL-MRDSA with PASL may insufficiently visualize the arteries distal to the stenosis and collateral circulation due to the RF saturation effect, but 4D-PACK, the novel technology of ASL-MRDSA using PCASL, can reveal these arteries, including delayed flow even at the late phases (Fig. 1 and Online Resource 1). In addition, ASL-MRDSA enables the creation of maximum intensity projection (MIP) images, consisting of all phases, by adding all phase data. MIP images of ASL-MRDSA with PCASL can depict vessels, including peripheral branches and the collateral circulations better than TOF-MRA based on our experience (Fig. 2). It has been reported that ASL-MRDSA with PASL can accurately evaluate the stage of moyamoya disease as well as DSA [26]. Furthermore, a comparison of ASL-MRDSA with PASL and ASL-MRDSA with PCASL in moyamoya disease reported that ASL-MRDSA with PCASL (4D-PACK) provided better visualization of the distal arteries and LMA collaterals, which was consistent with DSA [27]. Further, bypass surgery may be performed in cases of severe stenosis or occlusion of the major cerebral arteries. ASL-MRDSA can also assess the bypass and recipient arteries. Furthermore, labeling the bypass or its parent artery with a superselective labeling pulse enables the assessment of the bypass and recipient arteries in more detail (Fig. 3). A comparison between TOF-MRA and 4D-S-PACK indicated the superiority of 4D-S-PACK in depicting bypass patency and intracranial collateral circulation from the external carotid artery [28].

## Vascular malformation

**Fig. 4:**
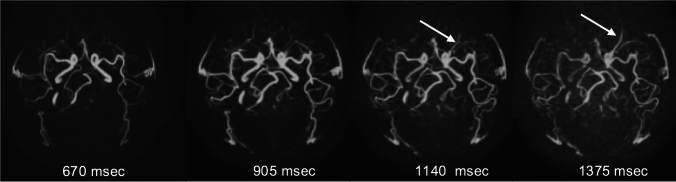
Left carotid-cavernous fistula. The axial views of mASTAR, one of ASL-MRDSA with PASL, are presented from the left to the right chronologically. mASTAR demonstrates an early depiction of the left superior ophthalmic vein (arrows). ASL-MRDSA: arterial spin labeling-based noncontrast magnetic resonance digital subtraction angiography, mASTAR: multi-TI MRA with ASTAR ASL technique, PASL: pulsed arterial spin labeling

**Fig. 5:**
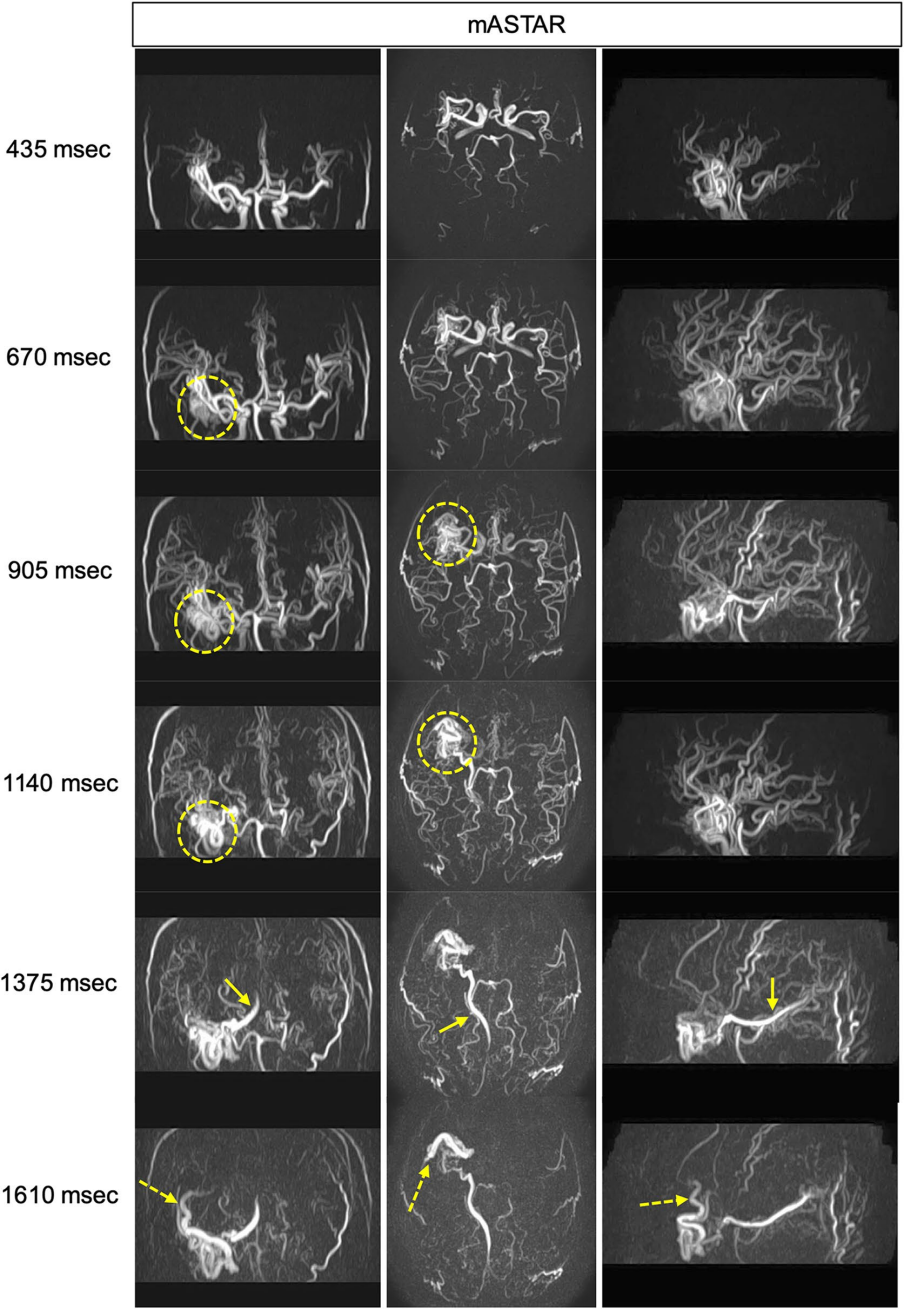
Arteriovenous malformation in the right temporal lobe. The coronal, axial, and sagittal views of mASTAR are shown sequentially from the left to the right columns. mASTAR demonstrates the nidus supplied by the middle cerebral arteries (yellow dotted circles), deep vein (yellow arrows), and Trolard vein (yellow dotted arrows) in that order. ASL-MRDSA with 3D acquisition can exhibit lesions in multiple directions. ASL-MRDSA: arterial spin labeling-based noncontrast magnetic resonance digital subtraction angiography, mASTAR: multi-TI MRA with ASTAR ASL technique

**Fig. 6:**
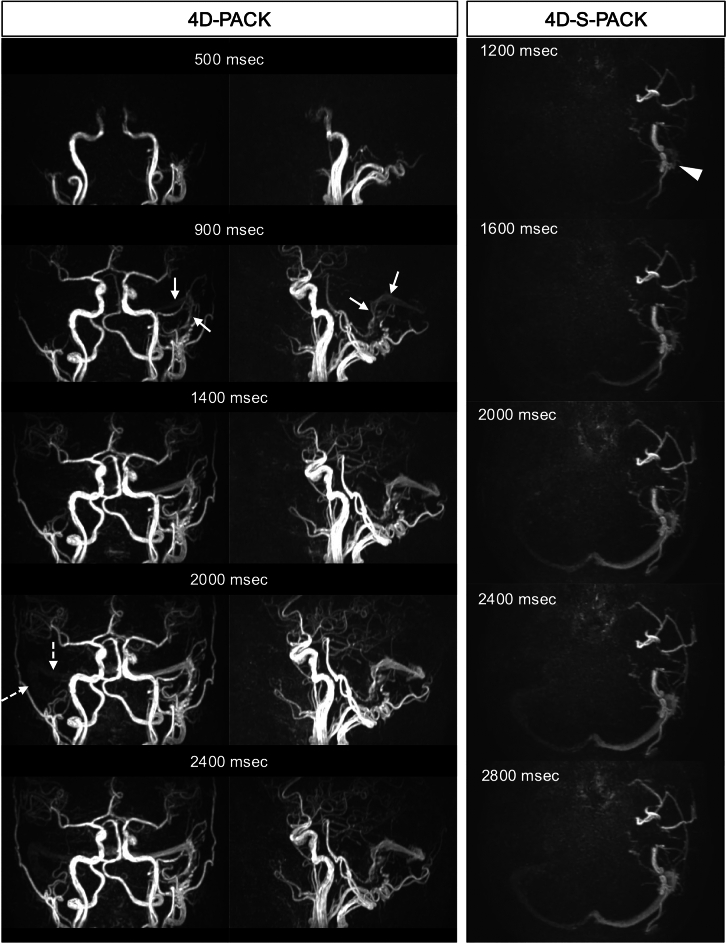
Dural arteriovenous fistula. The coronal and sagittal views of 4D-PACK and the axial views of 4D-S-PACK with the left external carotid artery labeling are shown in that order from the left to the right columns. 4D-PACK visualizes the shunt point at the left transverse-sigmoid sinus junction in the early phase (arrows), followed by gradual right transverse and sigmoid sinus visualization (dotted arrows). 4D-S-PACK reveals shunts from the left occipital and middle meningeal arteries to the left transverse-sigmoid sinus (arrowhead). 4D-PACK: 4D-MR angiography based on pseudo-continuous arterial spin labeling combined with CENTRA-keyhole and view-sharing, 4D-S-PACK: 4D-MR angiography based on super-selective pseudo-continuous arterial spin labeling combined with CENTRA-keyhole and view-sharing

The labeled spins are not maintained until the venous phase in ASL-MRDSA; thus, the veins are usually not visualized. However, the labeled spins immediately flow into the venous circulation in vascular malformations such as AVM, dural AVF, and carotid-cavernous fistula, enabling the visualization of draining veins [29]. Therefore, ASL-MRDSA can be used to evaluate the hemodynamics of various vascular malformations with comparable temporal resolution to DSA from any direction (Figs. 4, 5). Further, the superselective labeling pulse can label the feeding artery or its parent artery, enabling the hemodynamic assessments of each artery involved in vascular malformation such as DSA (Fig. 6 and Online Resource 2). ASL-MRDSA can reportedly determine the shunt location with good visualization of the main feeding arteries and venous drainage and successfully visualize cortical venous reflux in dural AVF [29, 30]. Furthermore, the use of superselective labeling pulses reduces overlap with other blood vessels, enabling the determination of more feeding arteries [31]. The usefulness of ASL-MRDSA with PASL for detecting AVM has also been demonstrated. However, ASL-MRDSA with PASL exhibits a limited depiction of the draining veins [32, 33]. This may be because ASL-MRDSA with PASL uses multiphase acquisition; therefore, the RF saturation effect occurs in the later phase, thereby decreasing intravascular signals. Therefore, ASL-MRDSA with PCASL using single time point acquisition, 4D-PACK, is superior to ASL-MRDSA with PASL for depicting the draining veins. Furthermore, combining a superselective labeling pulse has been useful for determining feeding arteries, accurately measuring the nidus, and correctly identifying venous drainage patterns [34]. Also, high-temporal resolution ASL-MRDSA may facilitate a more detailed hemodynamic assessments of complex vascular malformations.

## Giant cerebral aneurysm

**Fig. 7:**
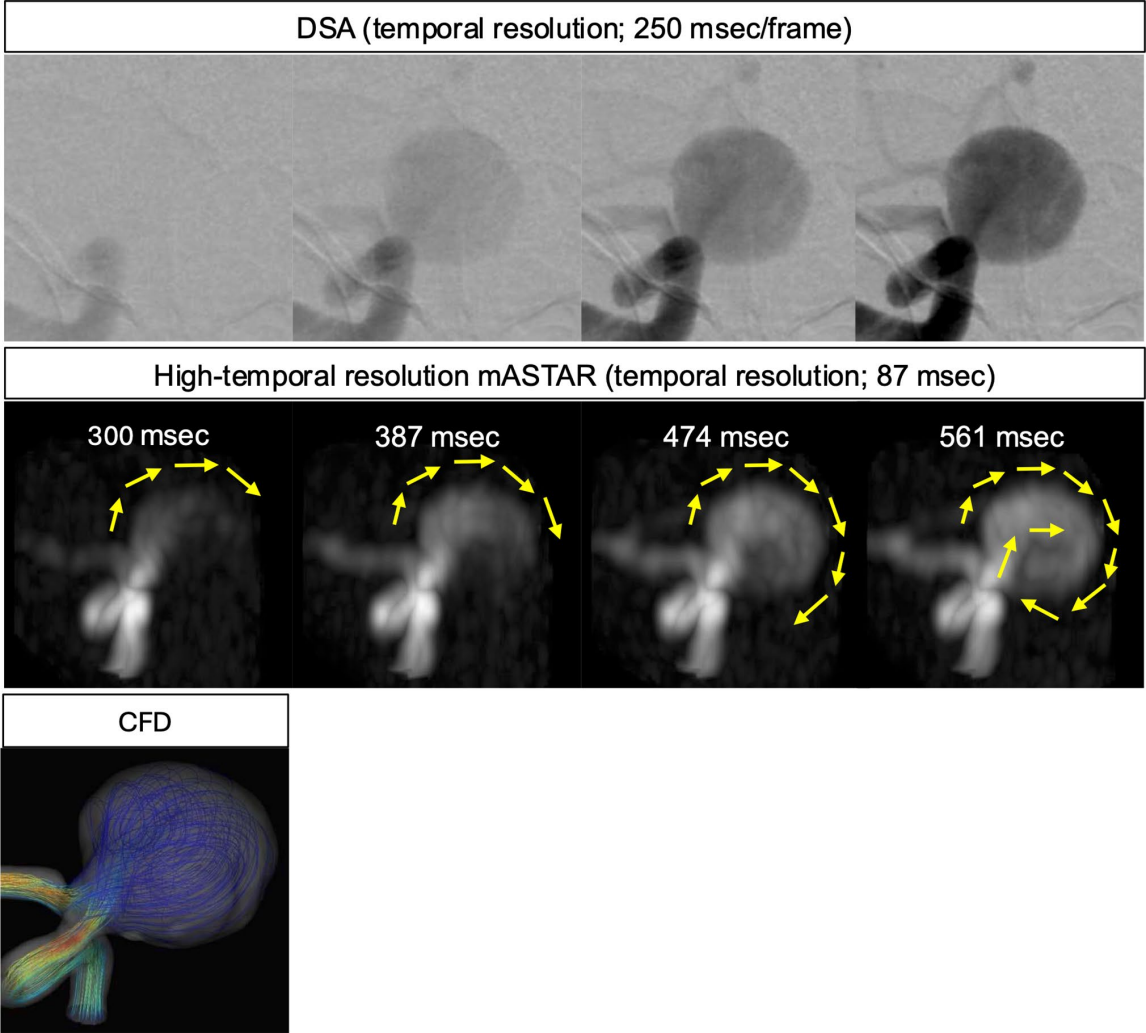
Giant cerebral aneurysm of the right internal carotid artery. DSA with the right internal carotid artery injection, high-temporal resolution mASTAR, and the computational fluid dynamics (CFD) image are shown in order from the upper to the lower columns. The high-temporal resolution mASTAR visualizes the vortex flow (yellow arrows) inside a giant cerebral aneurysm that is difficult to visualize with the temporal resolution of DSA. The vortex flow of high-temporal resolution mASTAR is similar to the jet streamlines of CFD. DSA: digital subtraction angiography, mASTAR: multi-TI MRA with ASTAR ASL technique

TOF-MRA is routinely utilized for the screening and follow-up of cerebral aneurysms. Assessing the entire aneurysm or its distal arteries may be difficult in cases of a giant cerebral aneurysm due to flow artifacts and the RF saturation effect. The hemodynamics of giant aneurysms and their distal arteries may be evaluated better than TOF-MRA because ASL-MRDSA is less affected by signal loss due to blood flow direction. Furthermore, the use of high-temporal resolution ASL-MRDSA, which demonstrates a much higher temporal resolution than DSA, enables a more detailed evaluation of the flow of labeled blood into a giant cerebral aneurysm [Gomyo M, et al. Frontier Technology in Non-Invasive Vascular Imaging; Arterial Spin Labeling-Based Non-Contrast MR Digital Subtraction Angiography on Cerebral Diseases. Presented at the 108th Scientific Assembly and Annual Meeting of the Radiological Society of North America (RSNA); November 26, 2023; Chicago, USA.] (Fig. 7 and Online Resource 3).

## Post-clipping and coiling surgery

**Fig. 8:**
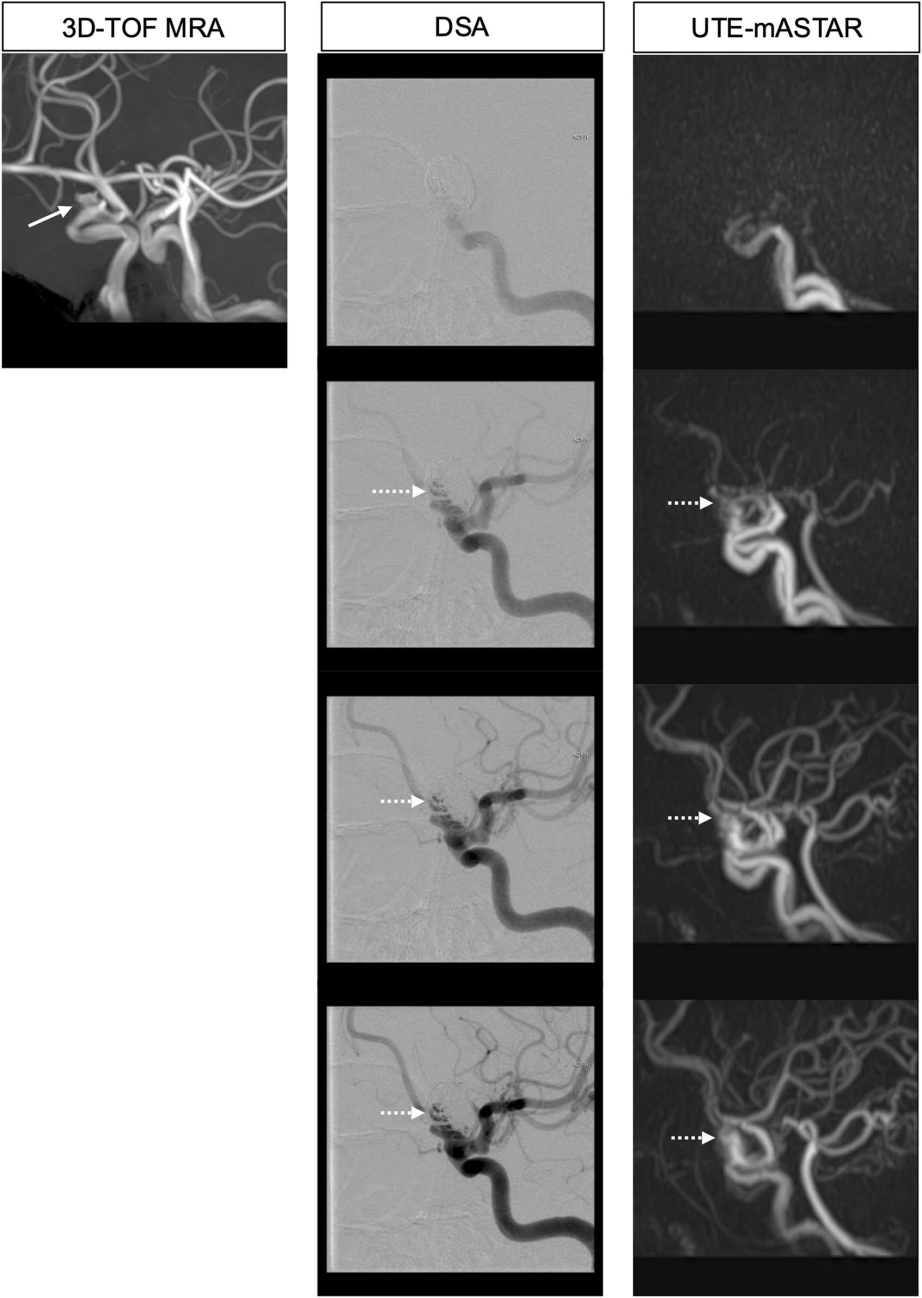
Post-coil embolization aneurysm of the right internal carotid artery. The oblique views of 3D-TOF-MRA, DSA with the right internal carotid artery injection, and UTE-mASTAR are presented from the left to the right columns in that order. 3D-TOF-MRA demonstrates only the neck of the residual aneurysm (arrow). UTE-mASTAR visualizes blood flow inside the residual lumen of the post-coil embolization aneurysm equivalently to DSA (dotted arrows). 3D-TOF-MRA: 3-dimensional time-of-flight magnetic resonance angiography, DSA: digital subtraction angiography, mASTAR: multi-TI MRA with ASTAR ASL technique, UTE: ultrashort-echo time

**Fig. 9:**
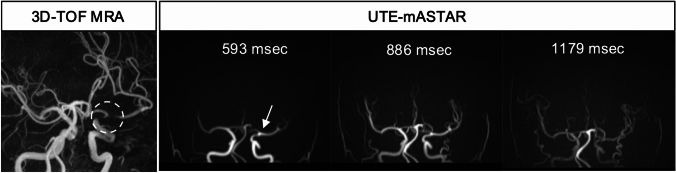
Post-clipping of the left internal carotid artery aneurysm. The artery’s signal loss (dotted circle) at the clipping site caused by magnetic susceptibility artifact is presented in 3D-TOF-MRA. UTE-mASTAR can assess that neither signal loss nor stenosis is present at the post-clipping site (arrow) and the hemodynamic information distal to the post-clipping site. 3D-TOF-MRA: 3-dimensional time-of-flight magnetic resonance angiography, mASTAR: multi-TI MRA with ASTAR ASL technique, UTE: ultrashort-echo time

TOF-MRA frequently faces difficulty in assessing the postoperative site due to signal loss caused by metal artifacts after clipping surgery or coiling with and without a stent assist of cerebral aneurysm. ASL-MRDSA with the UTE method is utilized to reduce signal loss due to metal artifacts by acquiring arterial signals before phase dispersion and is useful for assessing the persistence or recurrence of cerebral aneurysms and blood flow in an intracranial stent (Fig. 8). ASL-MRDSA with UTE also provides time-axis information, enabling the acquisition of hemodynamic information in addition to arterial morphology at the postoperative site and distal region (Fig. 9) [35].

## Brain tumor

**Fig. 10:**
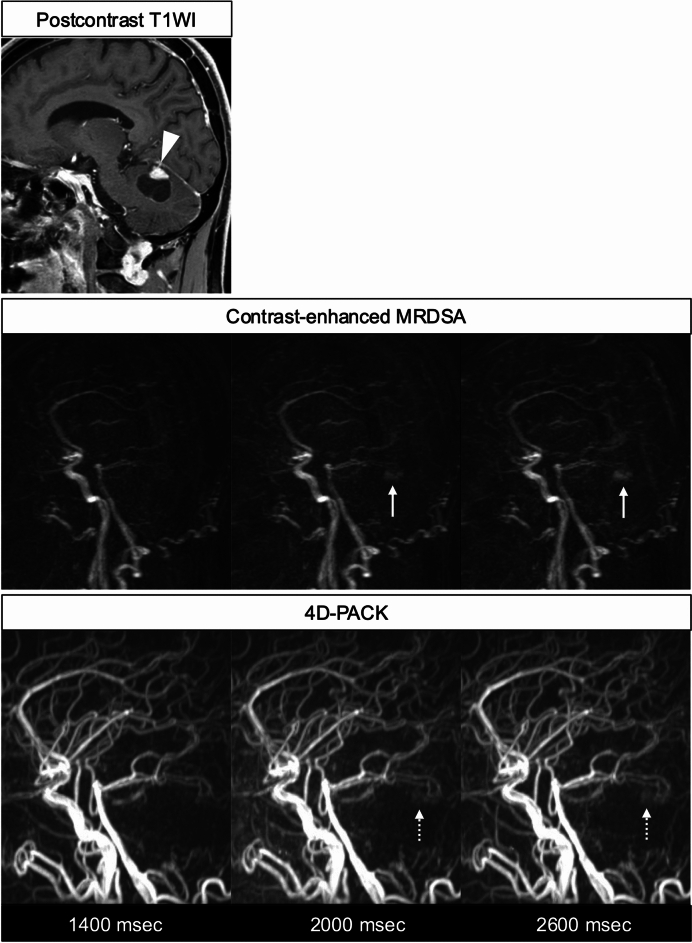
Hemangioblastoma. The postcontrast T1-weighted image (T1WI), contrast-enhanced MRDSA, and 4D-PACK are shown from the upper to the lower columns in that order. The postcontrast T1WI shows a cystic tumor with a mural nodule in the cerebellum (arrowhead). Contrast-enhanced MRDSA exhibits tumor enhancement corresponding to the nodule from the early arterial phases (arrows). 4D-PACK demonstrates a high signal that corresponds to the mural nodule, similar to contrast-enhanced MRDSA (dotted arrows). 4D-PACK: 4D-MR angiography based on pseudo-continuous arterial spin labeling combined with CENTRA-keyhole and view-sharing, MRDSA: magnetic resonance digital subtraction angiography

**Fig. 11:**
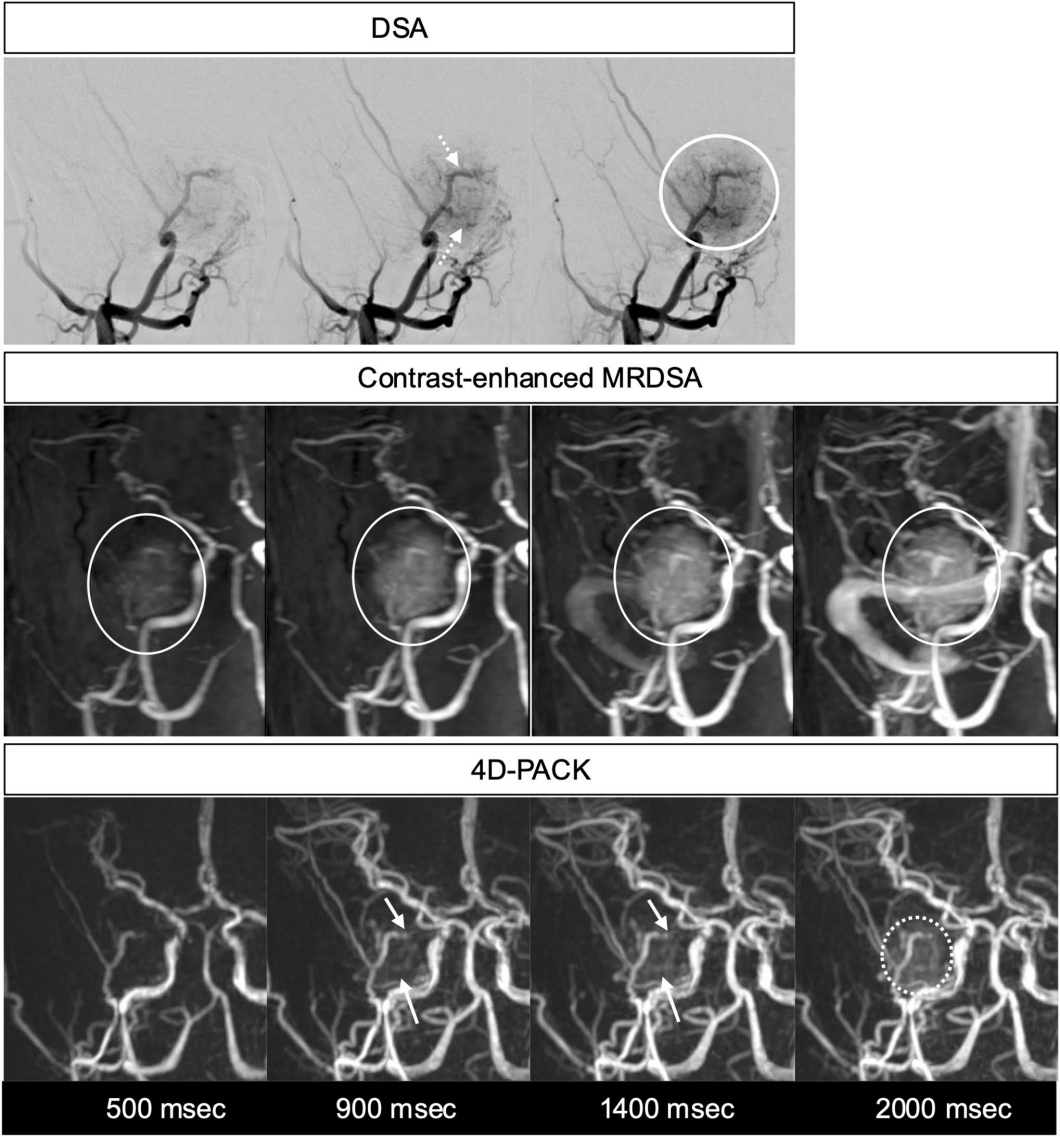
Angiomatous microcystic meningioma. DSA with the right external carotid artery injection demonstrates several tumor-feeding arteries branching from the external carotid artery (dotted arrows) and tumor stain (circle). The contrast-enhanced MRDSA clearly illustrates the tumor stain (circles), but identifying the tumor-feeding arteries is difficult. 4D-PACK visualizes the tumor-feeding arteries (arrows) and a tumor stain (dotted circle) such as DSA. 4D-PACK: 4D-MR angiography based on pseudo-continuous arterial spin labeling combined with CENTRA-keyhole and view-sharing, DSA: digital subtraction angiography, MRDSA: magnetic resonance digital subtraction angiography

The assessment of tumor vascularity is important for their differentiation and, in some tumors, for the decision to perform preoperative feeder embolization. On MRI, contrast-enhanced MRDSA is widely known as a method for assessing the hemodynamics of brain tumors. However, ASL-MRDSA can also depict tumor stains and feeding arteries in some hypervascular tumors [Gomyo M, et al. Evaluation of Intracranial Hypervascular Tumors Using ASL-based Non-contrast MRDSA. Presented at the 47th Annual Meeting of the Japanese Society of CNS Computed Imaging; April 19, 2024; Nagasaki, Japan] (Figs. 10, 11). Since ASL-MRDSA using PCASL can maintain high intravascular signals at late phases, tumor stains can be visualized more clearly. Furthermore, the use of superselective labeling pulses enables the assessment of hemodynamics in each parent artery of the tumor’s feeders.

## Pitfalls

ASL-MRDSA demonstrates the hemodynamics of various cerebral diseases. However, the signal duration of the labeled blood is limited even when using ASL-MRDSA with PCASL, making the visualization of all phases difficult, including veins. Performing a wide-range scan in a short time such as in contrast-enhanced MRDSA is also difficult. Therefore, ASL-MRDSA cannot visualize veins other than shunt veins in vascular malformations. It was reported that ASL-MRDSA cannot detect slow-filling residual AVM nidus in the confirmation of obliteration following gamma knife radiosurgery of AVM [36]. ASL-MRDSA also fails to visualize the findings corresponding to the slow-filling tumor stain observed in the contrast-enhanced MRDSA in brain tumors. In addition, ASL employs the subtraction process, which can frequently lead to issues such as image quality decrease and artifacts caused by patient’s motion.

## Discussion

Herein, we introduce the basic principles and the various novel techniques of ASL-MRDSA, which enables noninvasive hemodynamic assessment without a gadolinium-based contrast agent, and we demonstrate clinical applications for various cerebral diseases using ASL-MRDSA. TOF-MRA is routinely utilized for the noninvasive assessment of cerebral arteries using MRI, but ASL-MRDSA exhibits no signal loss due to blood flow direction and acquires images using a subtraction method. Therefore, ASL-MRDSA is superior to TOF-MRA in depicting peripheral branches and arteries with slow flow. In addition, ASL-MRDSA using the UTE method can assess the hemodynamics at the site and distal arteries after clipping or embolization, which is difficult to evaluate with TOF-MRA due to metal artifacts. However, ASL-MRDSA utilizes subtraction techniques to generate images. Hence, ASL-MRDSA makes the anatomical information assessment from the source images difficult, unlike TOF-MRA. Furthermore, as the spatial resolution of ASL-MRDSA is lower than that of TOF-MRA, we consider that TOF-MRA is currently necessary for the morphological evaluation of cerebrovascular lesions, and ASL-MRDSA remains a method to obtain additional hemodynamic information. PASL is a widely used labeling method in ASL-MRDSA, but the signal decreases at late phases caused by the RF saturation effect. Recently, ASL-MRDSA using PCASL has been developed, which has dramatically improved the ability to visualize peripheral branches and collateral circulations. Moreover, combining ASL-MRDSA using PCASL with superselective labeling pulses makes it possible to evaluate more detailed hemodynamic information of each major artery, similar to DSA. Contrast-enhanced MRDSA is predominantly used for hemodynamic assessment using MRI, but the temporal resolution of contrast-enhanced MRDSA is approximately 1 s even with high-speed scanning technology. Therefore, ASL-MRDSA, which exhibits a temporal resolution equivalent to that of DSA, is superior to contrast-enhanced MRDSA for assessing the hemodynamics of various vascular lesions in the arterial phases. In addition, the recently developed high-temporal resolution ASL-MRDSA using the fast 3D mode has the potential to assess the hemodynamics of complex vascular lesions and tumors feeding arteries in more detail. However, ASL-MRDSA does not allow full visualization, including veins and delayed tumor enhancement, as well as a wide scan area in a short time such as contrast-enhanced MRDSA. Useful hemodynamic information noninvasively in various cerebral diseases may be obtained by understanding the pitfalls and the characteristics of various scanning methods of ASL-MRDSA and applying them in clinical applications.

## Future outlook

The basic principles of ASL were introduced in the 1990s, but the widespread availability of high-magnetic-field MRI equipment and the development of novel technology have accelerated clinical application in recent years. In the future, ASL-MRDSA with PCASL, which allows for good visualization of distal small arteries and collaterals, is expected to play a crucial role as an indicator for monitoring the progress of cerebral artery stenosis and occlusive disease and for deciding on bypass surgery. However, the scan time for ASL-MRDSA with PCASL at our institution is approximately 8 min due to the protocol setting suitable for depicting the late phases, which is long for routine MRI. In particular, it demonstrates a crucial limitation for assessing acute cerebrovascular events. Similarly, reducing the total acquisition time is essential for its integration into routine clinical practice when labeling multiple arteries with superselective labeling pulses. In addition, improving the spatial resolution is another crucial challenge. ASL-MRDSA with UTE appears to have lower sharpness due to a radial scan; thus, achieving the spatial resolution comparable to TOF-MRA would enable the assessment of residual or recurrent aneurysms and branching arteries originating from postoperative sites, thereby serving as a powerful tool for the simultaneous assessment of vascular morphology and hemodynamics in the postoperative study. Addressing these issues may require artificial intelligence integration, which has demonstrated remarkable advancements in recent years. Furthermore, in recent years, the fully automatic segmentation of cerebral arteries using machine-learning has been attempted, and it has been reported that the temporal features of ASL-MRDSA play an important role in segmentation, including the distal small arteries [37]. If fully automated segmentation in ASL-MRDSA becomes clinically applicable, it may be possible to perform detailed evaluations of the hemodynamics of various vascular lesions, as well as the perfusion and watershed areas of each blood vessel, and blood flow modifications before and after treatment. Furthermore, the simultaneous acquisition of ASL-MRDSA and ASL-perfusion imaging using the time-encoded PCASL method is being developed, and if the clinical application of this scanning method becomes possible, it will become a powerful diagnostic tool for cerebrovascular diseases [38]. In the future, advancements in artificial intelligence and other technological innovations by various vendors are hoped to enable further clinical ASL-MRDSA applications.

## Conclusion

ASL-MRDSA is a noninvasive dynamic vascular imaging method without a gadolinium-based contrast agent, and recent remarkable technical developments have enabled its use for assessing various cerebral diseases.

## Supplementary Information

Below is the link to the electronic supplementary material.

Supplementary file1 Online Resource 1: Moyamoya disease with severe stenosis of bilateral M1 origin. 4D-TRANCE visualizes the collateral circulation from the anterior and the posterior cerebral arteries bilaterally. 4D-PACK visualizes the collateral circulation from the bilateral anterior and posterior cerebral arteries and peripheral branches of the bilateral middle cerebral arteries, which are difficult to visualize with 4D-TRANCE due to the radiofrequency saturation effects. Abbreviations___4D-TRANCE: 4D time-resolved angiography non-contrast-enhanced, 4D-PACK: 4D-MR angiography based on pseudo-continuous arterial spin labeling combined with CENTRA-keyhole and view-sharing (MP4 2017 KB)

Supplementary file2 Online Resource 2: Dural arteriovenous fistula. The coronal view of 4D-S-PACK with the left external carotid artery labeling, such as DSA, reveals shunts that exist at the left transverse-sigmoid sinus junction and its outflow into the right transverse and sigmoid sinus. Abbreviations___4D-S-PACK: 4D-MR angiography based on super-selective pseudo-continuous arterial spin labeling combined with CENTRA-keyhole and view-sharing, DSA: digital subtraction angiography (MP4 4427 KB)

Supplementary file3 Online Resource 3: Giant cerebral aneurysm of the right internal carotid artery. High-temporal resolution (HTR) mASTAR visualizes the vortex flow inside a giant cerebral aneurysm that is difficult to visualize with the temporal resolution of DSA. Abbreviations___DSA: digital subtraction angiography, mASTAR: multi-TI MRA with ASTAR ASL technique (MP4 2004 KB)
